# Ichthyosis1Read at the forty-second annual meeting of the Medical Association of Central New York, held at Auburn, October 19, 1909.

**Published:** 1910-10

**Authors:** A. A. Young

**Affiliations:** Newark, N.Y. 22 East Miller Street


### Ichthyosis[1]

By A. A. YOUNG, M.D.
Newark, N. Y.

THE writer's object in presenting this paper on ichthyosis is to produce an interchange of thought which shall tend to raise this affection above the idea now held of it as being an incurable disease, for which palliative treatment only can be rendered, hence none other is sought for. Such conclusion checks investigation and in like manner retards progress in etiological and therapeutical knowledge.

Since but little is known of ichthyosis, practically nothing of its etiology and of its pathology, the aid of fancy or of speculation must be invoked and substituted for the unknown. Such substitution must necessarily be here inserted. Fancy or imagination, is essential, is a necessity in the study of any and all "ologies"; without it chasms would remain unbridged and the unknown beyond, unexplored.

Macroscopically ichthyosis may be defined as a cutaneous affection appearing at or shortly after birth; frequently, but not always localised, consisting of horny epidermic scales, amphibian in appearance, varying in color from light to dark grey, dependent upon the thickness of the cuticle affected. I shall not take the time to refer to the numerous forms of ichthyosis as described in the various textbooks. It seems to me that the classification as given by most writers of ichthyosis congenita, ichthyosis hystrix, ichthyosis linearis, and the like, is absolutely valueless

---

1. Read at the forty-second annual meeting of the Medical Association of Central New York, held at Auburn, October 19, 1909.

if not misleading. There can be, barring a mixed infection, but one ichthyosis with a constant etiological factor, and a constant pathological condition.

I would not have it understood that ichthyosis is a hereditary disease, though it may be and doubtless is primarily congenital. Heredity, as I see it, for all microörganic infections is only the route over which is transmitted such life forces that end in reproduction of like organisms. The unanimity of observers points unmistakably to a microörganism and its transmissibility, and that such transmission takes place prior to the birth of the child or during its intrauterine life. Assuming that ichthyosis is transmissible, it is my belief that the morbific agent has its nidus within the genital tract and is transmitted directly from the mother to her unborn child.

Problematical also is the question, how the mother became ichthyotic and whether such ichthyotic condition is a local or constitutional condition. The inference to me is, that it is local because there are no constitutional symptoms apparent in an ichthotic mother; that the physiological action of the membrane of the genital tract has a "reverse action," which is a normal process, and thus conveying foreign and especially nonirritating material inward and upward, rather than downward and outward. If the microöorganism of ichthyosis be a nonirritant as it appears to be, then this peculiar but normal function of the membrane favors the entrance, the lodgement, and retention of this microörganism, and by its peculiar structure, the membrane is well fitted for the repose of the specific microörganism of ichthyosis in its chrysalitic life. It must be admitted that microorganisms as well as other creations, have their biological cycles; they must develop in accordance with fixed laws, a knowledge of which will materially aid in prophylaxis or successful treatment.

Anatomically the ichthyotic skin differs from the normal skin in that the papillæ are more or less hypertrophied with their conic apices considerably flattened; this flattening is evidently caused by the stratum of ichthyotic scales overlying the papillæ. The sebaceous and sudoriferous glands are diminished in size with a corresponding loss of function, and as a sequence there is a dry and scaly condition of the skin. It must be recognised that the ichthyotic scales differ in appearance from the normal epidermic scales, though the latter may be component parts of the former. What is believed to be another component part will be referred to a little later under etiology.

The functions of the papillæ in an ichthyolic skin, are apparently interferred with in proportion to their abnormal enlargement, and to the exudate covering them. Tactile sensations apparently are lost in proportion to the degree of development of the ichthyotic condition. The ichthyotic deposit upon the skin is doubtless a condition favored by the imperfect development of the sebaceous and sudoriferous glands, a nondevelopment that interferes with their normal functions. The nondevelopment of these glands may be due to mechanical agencies, may be due to several unknown conditions of which the presence of the specific microörganisms may be one. I believe, however, in a large measure, that nondevelopment is induced by mechanical conditions—namely, pressure upon these glands from the enlarged papillæ. It should be stated here that the diminutive size of the glands mentioned is not considered a true atrophy but an arrest of development, and that the enlarged papillæ not a true hypertrophy, but an overgrowth, giantism of practically normal tissue, a condition induced by microörganic involvement. Further, is it surmised that these conditions of the skin are produced during intrauterine life and that the infection inducing them at this time becomes inert and noncommunicable by direct contact after birth; that is, from child to child; and as has already been referred to that the ichthyotic agent, in order to perpetuate its existence, in carrying on its reproductive powers, must enter into the genitalia of another person, or perhaps of the child yet unborn, and be carried upward by the reverse action of the membrane finding lodgement as a chrysalis in the endometrium, and there await suitable conditions of pregnancy in order to complete its biological cycle.

The surmise of the source and biology of the infection is based upon the anatomical relation and physiological condition existing in an ichthyose; upon the fact that so far as known there is no involvement of other secretory or excretory organs of the body; that there is no systemic or hemic toxemia either in the mother or in the child; and that if death occurs it is shortly after birth and due to the nonexcretory or functionless condition of the skin itself. Of ichthyotic etiology but little can be said for practically nothing is known; but by analogy or inference from what is known of other transmissible affections, the etilogy of ichthyosis may be reasonably inferred.

That some of the contagious or transmissible affections are the products of microöorganic growths can no longer be doubted for such etiological factors have been isolated, observed, cultivated and tested. Since some of the transmissible affections are the products of known microörganic growths, it is reasonable to infer that all contagious and all of the so-called hereditary diseases are in like manner evolved though the specific microorganisms remain undiscovered.

I am aware that ichthyosis is classed by some of our best writers as a congenital deformity, or malformation, of the skin and not a disease. Such view, however, eliminates microörganisms as causal agents. In order to effect a deviation or change from normal conditions, or structures, to abnormal ones adequate causal agents there must be to produce such changes, otherwise the normal type will prevail. The very fact that the affection is hereditary, or transmissible, and contracted only during intrauterine life, indicates most clearly that such agents or forces are active, in producing dermal changes during gestation; and that such activity produces the macroscopic dermal conditions as become apparent in after life.

Viewed from the standpoint of etiology there must be etiological factors to produce the pathological conditions. Ichthyosis is a disease and not a deformity for there is both structural and functional changes in the skin, changes that are produced during intrauterine life and which so far as known is produced nowhere else. Ichthyosis may be a deformity, but it is an abnormality as well, an abnormality that begins only in early fetal life and is transmitted from a mother who to all appearances is infected prior to conception of the infected child, an infection which may even antedate her birth.

Briefly stated, my views which are almost entirely theoretical of the biology of ichthyosis are:

1. That there is a morbific agent, a specific microörganism, one that possesses the powers of reproduction.

2. That this morbific agent is dormant within and forms a part of the epidemic scale of the ichthyotic child.

3. That this infected scale with its vitalised organism is transmitted by some method to the female genitalia, where it becomes partially developed and later finds lodgement within the endometrium in a chrysalitic state.

4. That the condition of pregnancy favors the elimination of this chrysalis from its bed within the endometrium and aids in its deposition upon the skin of the fetus, where it is further developed and prepared for the office of reproduction, thus completing one biologic or life cycle.

For all microörganisms, as in other creations, the biological history of which is practically known, there must be in the beginning a germinal vesicle endowed with life, from which comes active development, and from such development a perfect organism,—an organism fitted for the deposition of other germinal vesicles thus perpetuating its life. It is with life forces and not inert matter that we largely deal, a fact too often the medical profession forgets. If this theory of the biology of micröorganisms be true, and it appears true in some forms of life, it may be asked, why are not all children born of an ichthyotic mother, ichthyotics?

From our present state of knowledge the only answer to be given is "because they are not," an illogical answer but one corresponding to fact. Immunity may exist for the reason that the fetal skin has a corresponding resisting power during fetal life, as normal skin has after birth; or it may be due to nonfertilisation; or it may be due to the absence of a culture medium; or it may be due in part to the protecting power of the vernix caseosa.

The opinion that ichthyosis is a neurosis as advocated by some writers seems incorrect from the fact that though the papillæ appear enlarged and distorted as they really are, yet the terminal nerve filaments in them remain unchanged. The conditions and phenomena connected with a case of ichthyosis seem only to warrant the conclusion that the affection is of microörganic origin, and that the specific microörganisms, like all microörganisms have a life growth a biologic cycle. It is assumed that ichthyosis is contracted only during early fetal life; that it is transmissible and transmitted only from the mother to her unborn child; that by direct contact of skin to skin it is never conveyed from individual to individual; that the conveyance must be by the way of the external genitalia, from thence carried to the endometrium; that it primarily affects the mother, secondarily the unborn child; that the morphology of the infecting agent is similar to that of all other micröorganisms capable of growth, capable of preservation, capable of reproduction.

Having stated, though imperfectly, my views relative to the etiology and pathology of ichthyosis, I will now give what appears to be an indicated treatment which treatment has a twofold object.

1. The destruction and elimination of the specific microorganisms of ichthyosis.

2. The restoration of the ichthyotic skin to normal skin structures so that its functions can be normally performed.

I have denominated the treatment about to be stated as an indicated treatment because, like the beginnings of ichthyosis it is still in embryo, and as such embryo it may expire before it is born. I cannot fortify my statements by a hundred or more consecutive cases, for I have had only one case for experimentation, and it being allowed by the family, it was conducted along the lines indicated and in accordance with the views I have stated here of etiology and pathology. Because recognised treatments were recognised failures, experimentation was asked for, allowed and adopted.

Having had considerable experience with formaldehyde as a therapeutic agent; having used it both as an external and internal medicament; knowing it to be one of our very best germicides; knowing it to be one of our best devitalisers of living tissues producing necrosis without decay; knowing it to be a devitaliser only of these tissues, or parts of tissues, with which it comes into direct contact, it was self-suggestive as a local therapeutic agent in ichthyosis, as an agent that would destroy the microörganisms in situ, cause exfoliation of affected cuticle sterilised; and by such exfoliation cause diminution in size of the affected papillæ without practically impairing their functions.

If the theory as heretofore stated be a fact, or approximately so, then the diminution in size of the papillæ will be followed by a corresponding increase in size of the sebaceous and sudoriferous glands, and especially so if properly nourished and stimulated to activity. A study of ichthyosis leads to the belief that the aforesaid glands are noninfected, and that their smallness is due largely to mechanical agencies from pressure. Another self-suggested medicament is pilocarpin, the physiological action of which makes it a valuable eliminant. It produces marked activity of the entire excretory glandular system with but little if any depression following such stimulation. In glandular activity, induced by pilocarpin, the sebaceous and sudoriferous glands show the most marked activity, an activity that increases their functions and causes an apparent decrease in size of the papillæ, a condition sought for. Having once stimulated abnormal or small glands, to activity, the tendency is toward increase till normality in size and function is reached.

Another action of pilocarpin should be considered and that is its power or influence in inducing and producing phagocytosis, a condition favorable to the elimination of foreign or abnormal agents whether animate or inanimate. Relative to treatment but little remains to be said. Oleaginous substances or medicaments, and "mechanical treatments," as are almost universally recommended, appear from testimony to be inert and worthless, at least their uses as curative agents have been confessed failures. For the reason that recognised treatments were recognised failures, by consent of parental authority an experimental treatment was adopted; and for reasons already stated pilocarpin, formaldehyde and juniper tar soap were selected.

The use of pilocarpin extended over a comparatively short period of time when by request it was discontinued, but such discontinuance I now consider illadvised because subsequent to such discontinuance the case made haste more slowly. The administration of the drug was so arranged as to produce at times profuse sweating and at other times a slightly discernable one.

Formaldehyde solution was used in varying strength from 1 to 10 to 1 to 500 dilution, the sensibility and appearance of the ichthyotic skin being the guide to percentages. The strength of formaldehyde solution can be determined only by the physician using it as he observes its action upon each individual patient. Experience here is the only teacher, not a past but a present one.

The use of juniper tar soap is considered advisable and especially so if the affected skin be covered with its lather and such lather allowed to remain for twelve hours and then removed with clear sterilised water. The method adopted for the treatment of the ichthyotic patient under observation was the use of sufficient pilocarpin to produce frequent and active glandular stimulation; pushing formaldehyde just to the devitalising stage of normal tissue, then stopping and waiting for exfoliation of abnormal epidermic tissue which was materially hastened by the use of juniper tar soap.

The patient, a moderately marked ichthyose, was first seen in the late summer of 1907, the entire skin then being moderately ichthyotic. Under treatment as outlined the ichthyotic condition had largely disappeared and is still disappearing and the indications point to a satisfactory recovery. Active treatment has been practically discontinued, only formaldehyde being used at long intervals. I have briefly stated my views of ichthyosis and the results of my first experimentation with it, and on account of its rarity it may be my last experimentation. If by the presenting of this paper I have assisted in any way in eliminating what may be a harmless though most unsightly and disagreeable affection, I am satisfied.

22 East Miller Street.

